# Proportion of Female Speakers at Academic Medical Conferences Across Multiple Specialties and Regions

**DOI:** 10.1001/jamanetworkopen.2020.18127

**Published:** 2020-09-28

**Authors:** Anuj Arora, Yuvreet Kaur, Fahima Dossa, Rosane Nisenbaum, Darby Little, Nancy N. Baxter

**Affiliations:** 1 Division of General Surgery, Department of Surgery, University of Toronto, Toronto, Ontario, Canada; 2 Li Ka Shing Knowledge Institute, St Michael’s Hospital, Toronto, Ontario, Canada; 3 Melbourne School of Population and Global Health, University of Melbourne, Melbourne, Victoria, Australia

## Abstract

**Importance:**

Women in medicine have been underrepresented at medical conferences; however, contributing factors have not been well studied.

**Objective:**

To examine the distribution of invited conference speakers by gender and factors associated with representation of women as speakers.

**Design, Setting, and Participants:**

This cross-sectional analysis used medical conference programs from March 2017 to November 2018 across 20 specialties in 5 regions (Australasia, Canada, Europe, the UK, and the US) that were obtained online or from conference conveners.

**Exposures:**

Gender of invited lecturers, panelists, and planning committee members for each conference based on name or picture and publicly available data on compositions of specialties by gender for included regions.

**Main Outcomes and Measures:**

Outcomes included the proportion of female speakers (invited lecturers and panelists), the number of single-gender panels, and the proportion of female speakers compared with the specialties’ gender composition. Correlations between the gender composition of conference planning committees and the proportion of female speakers were assessed. Multivariable regression models were used to evaluate factors independently associated with the proportion of female speakers at conferences.

**Results:**

A total of 8535 sessions (panels and invited lectures) with 23 440 speakers across 98 conferences were identified. Women accounted for 7064 (30.1%) of speakers; 1981 of 5409 panels (36.6%) consisted of men only, and 363 (6.7%) consisted of women only. The proportion of women speakers varied by region and specialty from 5.8% to 74.5%. In general, specialties with low baseline proportions of women (<20%) had a ratio of female speakers to female specialists greater than 1, whereas specialties with high baseline proportions of women (>40%) had a ratio of female speakers to female specialists less that 1. There was a strong positive correlation between the proportion of women on planning committees and conference representation of female speakers (*r* = 0.67; *P* < .001). The association remained statistically significant after controlling for other variables, including the regional gender balance of the specialty (odds ratio, 1.10; 95% CI, 1.04-1.15; *P* < .001 for every 10% increase in the proportion of women on the planning committee).

**Conclusions and Relevance:**

In this cross-sectional study, the proportion of female speakers at medical conferences was lower than that of male speakers, and more than one-third of panels were composed of men only. Increasing the number of women on planning committees may help address gender inequities.

## Key Points

**Question:**

What is the gender distribution of invited conference speakers, and what factors are associated with representation of women as speakers?

**Findings:**

In this cross-sectional analysis of 23 440 speakers at 98 conferences across 20 specialties between March 2017 and November 2018, 30.1% of speakers were women and 36.6% of panels were all-male panels. There was a significant positive correlation between the proportion of women on planning committees and representation of female speakers.

**Meaning:**

In this study, the proportion of female speakers was lower than that of male speakers and more than one-third of panels consisted of men only, suggesting that increasing the number of women on planning committees may help address gender inequities.

## Teaser

This cross-sectional study examines the proportion of female speakers at medical conferences and the factors associated with their representation as speakers overall, by specialty, and by region.

## Introduction

The number of women in medicine is increasing, but gender equity remains an issue at medical conferences.^1,2,3,4,5,6^ Speaker invitations enhance a presenter’s profile by increasing their visibility, raising their national and international presence, and providing opportunities for new collaborations and networks.^3^ These invitations are used as markers for promotion and establish individuals as experts.^7^ Gender disparities in representation as conference speakers can therefore substantially affect career progress for women, particularly in academics.^3,7,8,9^ The occurrence of all-male panels has become a symbol of gender inequity at conferences and has sparked a social media movement popularized on Twitter with *#manel*.

Previous studies have found that women are generally underrepresented as speakers in the fields of science, technology, engineering, and math.^8,9,10,11,12^ In medicine, this has been investigated in emergency medicine, critical care, global health, and surgery conferences, with similar findings.^4,5,6,8,10,11,12,13,14,15^ Even when women are well represented as conference speakers, there is attrition at more prestigious plenary talks. During the 2012 to 2016 European Association of Palliative Care conferences, women accounted for the majority of speakers but represented only 26.1% of plenary speakers.^7^ Previous studies of gender representation at medical conferences have limitations; many evaluated a single conference or focused on conferences within a single specialty or region.^4,5,6,8,10,11,12,13,14,15^ The most comprehensive study to date, by Ruzycki et al,^16^ assessed gender representation at 701 conferences over a 10-year period and found the proportion of women speakers in 2017 was only 34.1%. That study focused on conferences in Canada and the US and did not evaluate factors associated with greater representation of women.^16^

To better understand inequities in the representation of women at national medical conferences and to inform potential interventions, we conducted an international and multi-specialty analysis quantifying the proportion of women speakers at national conferences and evaluated factors independently associated with representation of women as conference speakers.

## Methods

For this cross-sectional study, we selected conferences from 5 regions that had English-language versions of scientific programs: Canada, the US, the UK, Europe, and Australasia. We next searched for major annual society conferences in 20 specialties (eAppendix 1 in the Supplement). The specialties were selected purposively to ensure gender composition variation of practitioners within the specialties. This was not considered human subjects research according to the government of Canada and therefore did not require ethics review.^17^ This study followed the Strengthening the Reporting of Observational Studies in Epidemiology (STROBE) reporting guideline.

Using a Google search conducted in Toronto, Canada, including the keywords “[region] [specialty] conference” (eAppendix 1 in the Supplement), we identified the main conference held during 2018 for each specialty within each region. For example, the main conference for cardiology in Canada was determined by searching “[Canadian] [Cardiology] Conference,” resulting in “Canadian Cardiovascular Congress 2018” as the first option. If multiple conferences were identified, we selected the conference associated with the region’s largest specialty association. In cases in which the 2018 conference program had not been finalized at the time of the search, the 2017 program was used. The search included conference programs from March 2017 to November 2018.

The program for each included conference was obtained from the conference or society website. If the program was not available online, we contacted the organizing committee and followed up via email and telephone if necessary. If we were not able to obtain a copy of the program, an alternative conference was selected from the region.

### Conference Data Extraction and Session Categorization

From each program, we categorized conference sessions as invited lectures (sessions with a single speaker) and panels (sessions with multiple speakers). We excluded pre- and postconference workshops and courses, poster presentations, case presentations, and oral presentations of research abstracts.

For each speaker, we extracted the first and last name and assigned a binary definition of gender (man or woman) based on names or pictures provided in the conference program. In cases of gender-ambiguous names, we searched online for pictures or institutional profiles of the named individuals. If online profiles could not be found and pictures were not available, we recorded the gender most commonly associated with the individual’s name using an online tool.^18^ This program uses Google's database to analyze the first name to determine whether a name is more common for a man or a women. For conferences for which only first name initials were provided, we searched for the presenter’s online, institutional, or PubMed profile to find their first name. If this was unsuccessful, the name was recorded as missing and excluded from our analysis. For conferences with more than 50 sessions of a single type on a single day, we randomly sampled 50 sessions from that category on that day. Speakers who presented more than once within a single session were only counted once for that session but would be recorded multiple times if presenting in different sessions at the same conference.

For each conference, we identified members of the conference planning committees, and using the same name- and photo-based strategy, we determined the gender composition of the committee. If conference planning committee data were not available in the online program, we emailed or called the conference or society contact to obtain this information. If there was no response, we attempted to contact the society president. If still unavailable, this information was recorded as missing.

### Quality Control

Each program was reviewed by 1 of 3 extractors (A.A., Y.K., or D.L.). A 10% random sample of the database was reviewed by a second extractor to check for errors in data entry and classification before analysis. Any discrepancies were resolved by discussion. The Cohen κ statistic (with 95% CI) was calculated for the extraction audit between raters.^19^

### Gender Composition of Specialties

We obtained gender composition of medical specialties for each region using regional data registries.^20,21,22,23,24,25^ Aggregate data for Europe were unavailable; however, the gender composition of medical specialties was available for 6 countries (the Czech Republic, Finland, France, Germany, Switzerland, and the UK), representing 45% of the physicians in Europe.^22,23,26,27,28,29,30^ By pooling the number and gender of physicians in each specialty from these 6 countries, the gender compositions for European specialties were estimated.

### Statistical Analysis

We used descriptive statistics to evaluate the number and proportion of female speakers (panelists and invited lecturers) and single-gender panels (all-male and all-female panels). For each region, we calculated the Spearman correlation coefficient between the proportion of women on program committees and the proportion of female conference speakers. Eighteen conference planning committees were missing; thus, these conferences were excluded from this analysis. We considered the strength of association moderate if the absolute *r* value was between 0.40 and 0.59, strong if the absolute *r* value was between 0.60 and 0.79, and very strong if the absolute *r* value was between 0.80 and 1.00.^31^ A bubble plot was created to illustrate the ratio of the percentage of female speakers in each conference to the percentage of female specialists in that respective region. A multivariable beta regression model with a logit link^32^ was used to assess the associations between the proportion of female speakers in conferences (0%-100%) and region of the conference (Australasia, Canada, Europe, the UK, and the USA [reference]), the percentage of women on planning committees (0%-100%), the percentage of women in the specialty within the region (0%-100%), and total number of sessions at each conference. This model allowed for the estimation of odds ratios (ORs) and 95% CIs. Goodness of fit was determined if the ratio of the Pearson χ^2^ statistic to the number of degrees of freedom was approximately 1. Statistical analyses were performed using SAS software, version 9.4 (SAS Institute Inc). All *P* values were 2-sided, and *P* < .05 was considered statistically significant.

## Results

We identified 98 conferences held during 2017 to 2018 (eAppendix 1 in the Supplement), representing 20 specialties (2 specialty conferences were combined in Canada). In total, there were 9536 sessions, with a range of 3 to 625 sessions per conference. We evaluated the gender distribution of speakers for all sessions at 91 conferences (n = 6419) and 67.9% of sessions at 9 larger conferences with more than 50 sessions per day (n = 2116), resulting in a final sample of 8535 sessions, including 5409 panels and 3126 invited lectures. Three conferences had no invited lectures, and 1 conference had no panels. A total of 126 speakers (0.005%) were excluded from all analyses because the gender could not be identified for at least 1 panelist using our search strategy. For 51 speakers, we determined gender based on common use. Interrater reliability for the data extraction audit was high between all 3 rater pairs (κ > 0.97).

**Figure 1.**
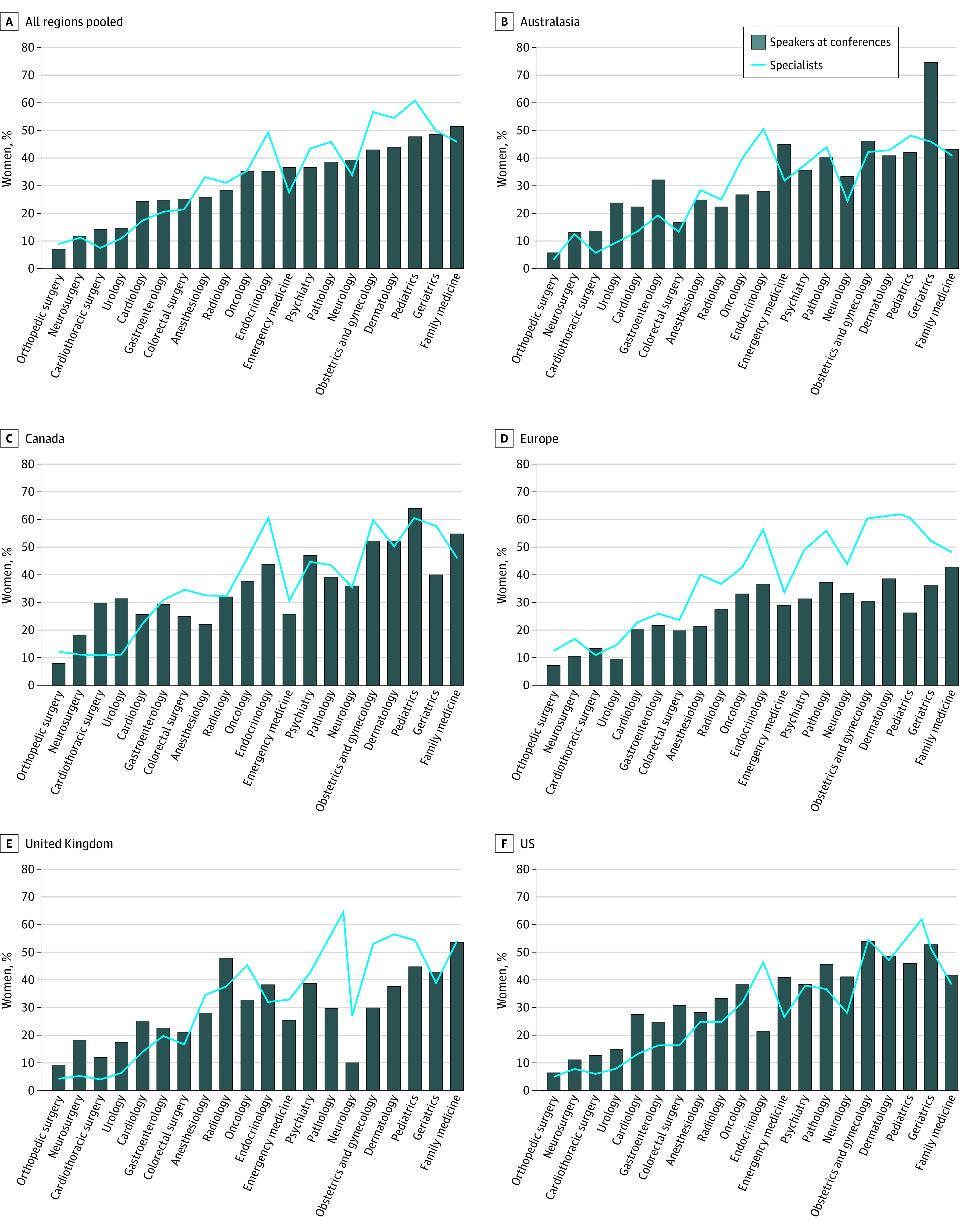
Percentage of Women as Speakers at Conferences and in Each Specialty

We identified a total of 23 440 speakers, including 7064 female speakers (30.1%), with a median of 30.5% female speakers (interquartile range [IQR], 21.6%-40.6%; 95% CI, 28.4%-33.8%) at each conference. The median gender distributions of speakers for invited lectures (25.0% women; IQR, 6.3%-39.4%; 95% CI, 22.0%-31.5%) and panels (31.3% women; IQR, 20.2%-39.5%; 95% CI 28.6%-34.6%) were similar. Representation of women at conferences ranged widely; at 6 conferences, less than 10% of speakers were women, and at 2 conferences, more than 60% of speakers were women (Table 1). In general, the pooled data from all regions showed that specialties with lower proportions of women had higher-than-expected proportions of female speakers, and specialties with higher proportions of women had lower-than-expected proportions of female speakers (Figure 1). This general trend remained consistent in each individual region.

**Table 1.** Conference Data by Region Clustered by Proportion of Female Speakers

Region	OS	NS	CTS	Uro	Cardio	GE	CRS	Anes	Radio	Onc	Endo	EM	Psych	Path	Neuro	OB/GYN	Derm	Ped	Ger	FM
Australasia																				
Women in specialty, %	3.2	12.5	5.7	9.5	13.4	19.3	13.3	28.3	24.9	40	50.5	31.8	37.7	43.9	24.4	42.2	42.8	48.1	45.9	40.8
Female speakers, %	5.8	13.2	13.6	23.7	22.3	32.1	16.7	24.8	22.3	26.7	28	44.8	35.6	40.1	33.3	46.2	40.8	42	74.5	43.2
All-male panels, No. (%)	28 (82.4)	5 (55.6)	8 (36.4)	7 (29.2)	23 (41.1)	12 (27.2)	3 (33.3)	11 (33.3)	23 (60.5)	3 (37.5)	3 (50.0)	1 (2.0)	15 (31.9)	15 (35.7)	2 (20.0)	1 (10.0)	1 (3.8)	3 (30.0)	0	9 (45.0)
All-female panels, No. (%)	0	0	0	1 (4.2)	1 (1.8)	2 (4.5)	0	0	2 (5.3)	0	0	1 (2.0)	5 (10.6)	2 (4.8)	0	1 (10.0)	0	0	7 (53.9)	2 (10.0)
Women on PC, No. (%)	NR	NR	3 (20.0)	3 (50.0)	0	3 (33.3)	0	NR	3 (30.0)	1 (12.5)	3 (42.9)	NR	2 (22.2)	31 (45.6)	NR	8 (50.0)	3 (21.4)	NR	9 (56.3)	3 (60.0)
Canada																				
Women in specialty, %	12.3	11.1	10.9	11.2	22.1	30.9	34.6	32.6	32.2	46.1	60.5	30.6	44.7	43.6	35.5	59.7	50.2	60.5	57.6	46
Female speakers, %	8	18.2	29.8	31.4	25.6	29.3	25	21.9	32	37.5	43.8	25.8	47	39.1	35.9	52.2	52	64	40	54.8
All-male panels, No. (%)	26 (70.3)	5 (50.0)	0	10 (76.9)	26 (37.1)	24 (40.7)	1 (33.3)	18 (60.0)	2 (14.2)	1 (100)	4 (13.3)	3 (60.0)	4 (25.0)	4 (30.8)	0	7 (21.9)	3 (15.8)	2 (4.7)	5 (45.5)	21 (14.3)
All-female panels, No. (%)	0	0	0	0	2 (2.9)	2 (3.4)	0	1 (3.3)	0	0	4 (13.3)	0	1 (6.3)	2 (15.4)	0	8 (25.0)	1 (5.3)	16 (37.2)	0	32 (21.8)
Women on PC, No. (%)	7 (35.7)	0	7 (33.3)	NR	13 (39.4)	3 (25.0)	1 (16.7)	0	10 (62.5)	2 (22.2)	6 (37.5)	5 (33.3)	NR	0	8 (53.3)	15 (57.7)	6 (66.7)	12 (85.7)	3 (33.3)	7 (58.3)
Europe																				
Women in specialty, %	12.5	16.8	10.9	14.6	22.8	26	23.6	40	36.7	42.9	56.3	33.6	48.9	56	43.9	60.4	61.9	60.6	52.2	48
Female speakers, %	7.2	10.4	13.3	9.3	20.2	21.6	19.7	21.5	27.5	33.2	36.6	28.9	31.3	37.2	33.3	30.3	38.6	26.3	36.1	42.9
All-male panels, No. (%)	57 (71.3)	34 (72.3)	14 (63.6)	103 (74.6)	105 (47.9)	34 (24.6)	8 (47.1)	58 (53.7)	85 (39.0)	27 (25.0)	12 (28.6)	20 (37.7)	22 (32.4)	11 (14.9)	12 (20.0)	8 (25.0)	34 (20.0)	2 (40.0)	8 (21.1)	0
All-female panels, No. (%)	0	0	2 (9.1)	0	3 (1.4)	2 (1.4)	0	3 (2.8)	9 (4.1)	2 (1.9)	4 (9.5)	2 (3.8)	2 (2.9)	0	3 (5.0)	0	5 (2.9)	0	1 (2.6)	0
Women on PC, No. (%)	1 (4.0)	3 (10.7)	2 (12.5)	NR	16 (17.8)	6 (25.0)	1 (25.0)	11 (16.9)	7 (25.9)	100 (33.3)	8 (30.8)	8 (21.6)	6 (30.0)	6 (28.6)	7 (33.3)	NR	7 (46.7)	1 (12.5)	23 (34.3)	10 (52.6)
UK																				
Women in specialty, %	4.2	5.3	4	6.4	14	19.7	16.7	34.5	37.6	45.3	32	32.9	42.6	64.5	27	52.9	56.5	54.2	38.8	54
Female speakers, %	9	18.2	11.9	17.4	25.1	22.6	20.9	28	47.8	32.7	38.3	25.4	38.6	29.7	10	29.8	37.5	44.8	42.9	53.5
All-male panels, No. (%)	36 (75.0)	3 (60.0)	26 (70.3)	19 (55.9)	23 (35.4)	16 (30.8)	7 (33.3)	0	0	8 (26.7)	8 (25.8)	2 (40.0)	17 (26.2)	6 (35.3)	3 (60.0)	16 (31.4)	6 (24.0)	6 (21.4)	2 (11.8)	4 (9.3)
All-female panels, No. (%)	1 (2.1)	0	1 (2.7)	0	3 (4.6)	1 (1.9)	0	0	1 (9.1)	1 (3.3)	1 (3.2)	0	6 (9.2)	0	0	3 (5.9)	1 (4.0)	4 (14.3)	0	6 (14.0)
Women on PC, No. (%)	NR	1 (12.5)	7 (23.3)	0	19 (30.6)	NR	3 (25.0)	0	4 (50.0)	17 (47.2)	10 (46.7)	4 (66.6)	1 (25.0)	NR	3 (30.0)	5 (36.0)	57 (52.8)	NR	5 (38.5)	NR
US																				
Women in specialty, %	5	7.8	6	8	13.2	16.4	16.4	24.9	24.7	31.9	46.4	26.6	38	36.7	28.1	54.5	47.1	61.9	51.2	38.4
Female speakers, %	6.5	11.2	12.7	14.8	27.5	24.8	30.8	28.3	33.3	38.2	21.3	40.9	38.3	45.6	41.1	54	48.5	45.9	52.8	41.8
All-male panels, No. (%)	160 (83.3)	42 (63.6)	22 (64.7)	77 (72.0)	39 (22.3)	77 (41.4)	14 (31.8)	79 (38.5)	2 (16.7)	39 (27.9)	6 (40.0)	13 (22.8)	19 (31.7)	13 (22.8)	104 (31.2)	6 (13.6)	29 (14.4)	27 (28.4)	5 (9.8)	7 (31.8)
All-female panels, No. (%)	1 (0.1)	0	0	4 (3.7)	3 (1.7)	8 (4.3)	1 (2.2)	17 (8.3)	0	9 (6.4)	0	8 (14.0)	9 (15.0)	11 (19.3)	48 (14.4)	19 (43.2)	23 (11.4)	24 (25.3)	11 (21.6)	7 (31.8)
Women on PC, No. (%)	2 (10.5)	4 (21.1)	0	8 (11.1)	55 (35.5)	NR	27 (38.6)	NR	2 (22.2)	66 (40.5)	8 (50.0)	5 (26.3)	1 (100)	9 (50.0)	5 (71.4)	5 (45.5)	7 (46.7)	12 (46.2)	NR	2 (40.0)

Abbreviation: Anes, anesthesiology; Cardio, cardiology; CRS, colorectal surgery; CTS, cardiothoracic surgery; Derm, dermatology; EM, emergency medicine; Endo, endocrinology; FM, family medicine; GE, gastroenterology; Ger, geriatrics; Neuro, neurology; NS, neurosurgery; NR, not reported; Radio, radiology; OB/GYN, obstetrics and gynecology; Onc, oncology; OS, orthopedic surgery; Path, pathology; PC, planning committee; Ped, pediatrics; Psych, psychiatry; Uro, urology.

**Figure 2.**
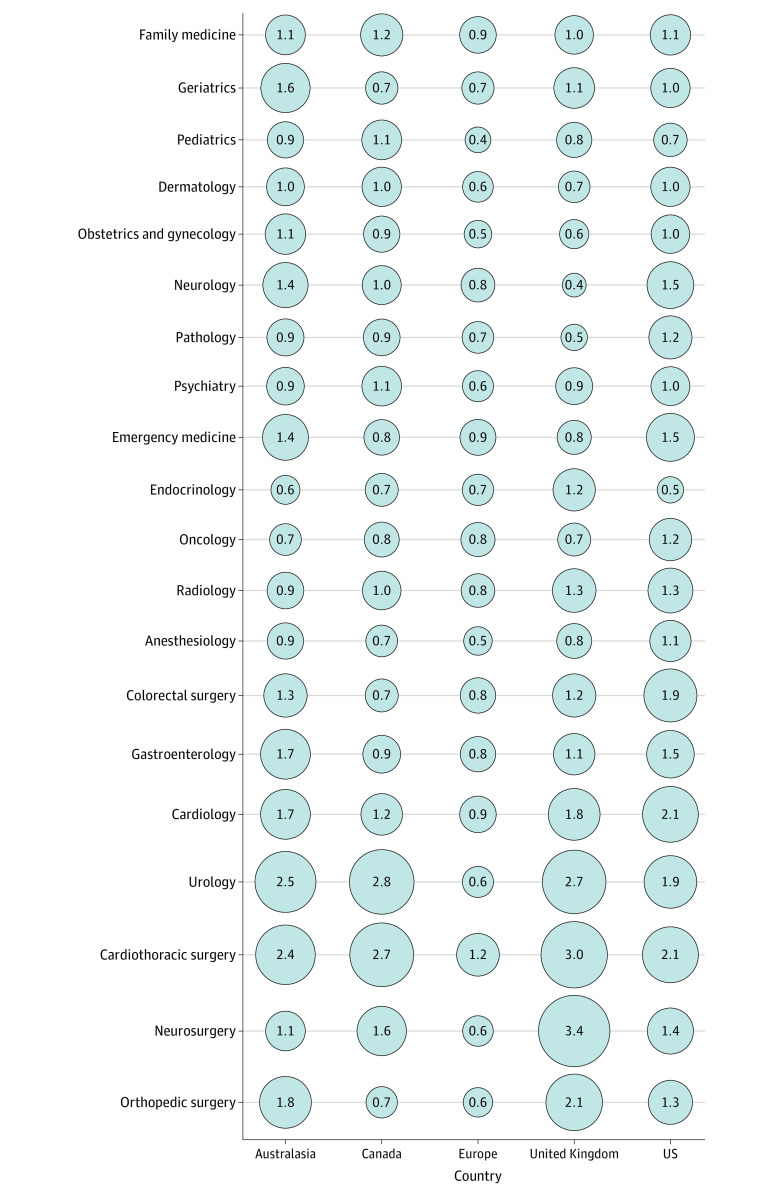
Bubble Plot of the Ratio of the Proportion of Female Speakers at Each Conference to the Proportion of Female Specialists in Each Respective Specialty by Region The size of the bubble reflects the size of the ratio between speakers and baseline specialists. A small bubble reflects a low proportion of female speakers relative to the proportion of women in the specialty, and a large bubble reflects a high proportion.

Representation of women as speakers also varied by region (Figure 2). In Europe, the proportion of female speakers was generally lower than the baseline proportion of women in the specialty. In contrast, in the US, the proportion of female speakers was generally higher.

Of conferences with panels, we identified 1981 all-male panels (36.6% of all panel sessions). Only 4 conferences did not have any all-male panels. In contrast, there were 363 panels with only female speakers (6.7% of all panels) and 39 conferences without an all-female panel. Only 1 conference had no single-gender panel (European Family Medicine), but this conference had only 2 panel sessions.

**Figure 3.**
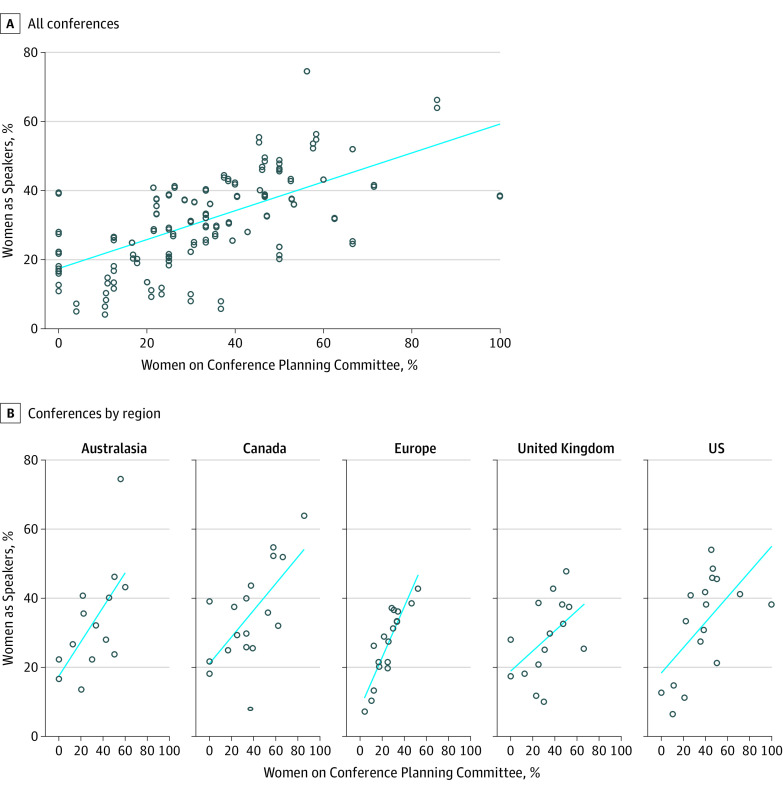
Association Between Gender Balance in Organizing Committees and Speaker Sex Ratio Using the Spearman Rank Correlation Coefficient The circles represent each conference, and the line indicates the correlation.

We identified the planning committees for 82 conferences; in 3 cases, the committees had only 1 individual listed. The median proportion of women with steering committee membership was 33.3% (IQR, 20.8%-46.7%). Men accounted for more than 50% of steering committee members at 69 of 82 conferences (84%). We found an overall strong positive correlation between the proportion of women on steering committees and representation of female speakers at conferences (*r* = 0.67; *P* < .001), with variability in the strength of the correlation by region (*r* = 0.56 to *r* = 0.90; *P* = .02 to *P* < .001) (Figure 3).

The multivariable beta regression model fit the data well (Pearson χ^2^ / degrees of freedom, 1.03). Factors associated with the proportion of female speakers at conferences in our analysis included the percentage of women specialists within the specialty in the respective region (OR, 1.28; 95% CI, 1.21-1.36; *P* < .001 for every 10% increase), the percentage of women on the planning committee (OR, 1.10; 95% CI, 1.04-1.15, *P* < .001 for every 10% increase), and region (Table 2); compared with the US, the odds of female speakers in Europe was 28% lower (OR, 0.72; 95% CI, 0.55-0.95, *P* = .02). All other regions were similar to the US.

**Table 2.** Multivariable β Regression of Factors Associated With the Proportion of Female Speakers at Conferences

Factor	Odds ratio (95% CI)	*P* value
No. of sessions	1.000 (0.999-1.002)	.37
Percentage of women on planning committee (10% increments)	1.095 (1.044-1.149)	<.001
Percentage of women in specialty (10% increments)	1.280 (1.207-1.359)	<.001
Region		
US	1 [Reference]	
Australasia	1.164 (0.874-1.551)	.29
Canada	1.001 (0.765-1.310)	.99
Europe	0.718 (0.545-0.945)	.02
UK	0.899 (0.673-1.201)	.47

## Discussion

In this cross-sectional analysis, men outnumbered women as invited lecturers, panelists, and planning committee organizers in most conferences by an approximate ratio of 2:1. Women accounted for less than 50% of speakers at 92 of the 98 conferences sampled. Approximately one-third of panels at conferences were male only, and only 4 conferences did not have any male-only panels. Female representation at conferences varied by region; in Europe, the proportion of female speakers was generally lower compared with the proportion of women in the specialty. Univariable analysis showed a strong positive correlation between the proportion of women on steering committees and representation of female speakers at conferences. The multivariable analysis indicated that the proportion of women on the planning committee was significantly associated with the proportion of women as speakers at the respective conferences, even after adjustment for other factors, including the proportion of women in a given specialty and region.

Gender equity at conferences has been evaluated in scientific and medical conferences for individual conferences, individual disciplines and specialties, and different countries.^4,5,6,8,10,11,12,16^ Our finding that women represented 30.1% of speakers overall is consistent with the results of a comprehensive review by Ruzycki et al^16^ of 701 medical and surgical conferences in Canada and the US in which the proportion of female speakers was only 34.1% and with studies in critical care, emergency medicine, and surgery that reported a proportion of female speakers globally of 15% to 35%.^4,5,6,13,14^ Our study expands on these previous findings by including surgical and medical specialties across 5 regions and found independent factors associated with the proportion of female speakers at conferences.

Our study is unique in that it quantified the proportion of single-gender panels in a contemporaneous sample of medical conferences. Johnson et al^12^ reported that, at psychology conferences over a 13-year period, the prevalence of all-male panels was 15.5% (129 sessions) compared with 36.6% (1981 sessions) found in our study. Among a sample of 21 surgical conferences, the prevalence of all-male panels was even higher (39.5%; 893 sessions).^4^ A study by Casadevall and Handelsman^33^ found that the gender of the panel convener was associated with the gender composition of a panel, with panel sessions with men conveners having fewer female presenters compared with panel sessions with female conveners. This finding was also mirrored in an analysis from the Society of American Gastrointestinal and Endoscopic Surgeons conference between 2009 and 2018. For sessions that had no female convener, 52% of panels were all male. For sessions that had at least 1 female convener, 19% of panels were all male.^6^ We were unable to perform a similar analysis because in the conferences that we sampled, there was variability in the presence of a convener; however, this may be a potential strategy to decrease the number of all-male panels.

Our study is one of the largest to compare geographic differences in the proportion of women as speakers in a variety of specialties with the proportions of women in those specialties. This strategy has been used to examine the representation of women as speakers at emergency medicine, critical care, and surgical conferences.^4,13,14^ Carley et al^14^ found that the proportion of speakers in emergency medicine matched the proportion of women in the specialty and suggested that the conference gender balance was associated with the workforce rather than with conference selection bias. Mehta et al^13^ used an identical approach for critical care and concluded that a speaker gender gap existed. Gerull et al^4^ compared the surgical society membership composition with the speaker composition and found that women were underrepresented as speakers, especially in plenary speaking roles. Our study showed that the proportion of women as speakers was not always balanced with the proportion of female specialists in different specialties and regions. Although we did not explore reasons for regional differences, we expanded on previous findings^4,13,14^ by showing that, independent of the proportion of women across specialties and regions, the number of women on the planning committee was associated with the proportion of female speakers at a conference.

Understanding factors associated with the speaker gender proportion is important. Carley et al^14^ suggested that the proportion of speakers in emergency medicine is associated with the workforce. Lithgow et al^34^ expanded on this finding by analyzing 108 medical and surgical conferences from Canada and the US and observed that specialty composition and the proportion of women on planning committees were associated with increased proportions of female speakers. Our multivariable analysis demonstrated a similar association in all regions studied; the association between the number of women on planning committees and representation of women as speakers was generalizable across specialties and countries. Johnson et al^12^ noted that an increase the number of speaker slots was not associated with an increase the number of female speakers at a psychology conference. Similarly, we did not find that the number of speaker sessions was associated with the proportion of female speakers. The region of the conference was also associated with speaker gender balance, with conferences in European conferences having 28% lower odds of having female speakers compared with US conferences.

### Strengths and Limitations

This study has strengths. We assessed gender balance of speakers on a large scale across a variety of specialties in medicine and surgery and in multiple regions, providing a comprehensive and contemporary overview of the representation of women at major conferences. This allows the results to be generalizable and reflective of the medical profession as a whole. We also studied factors associated with gender distribution at these conferences, and using multivariable analysis, we identified increasing the proportion of women on planning committees as an actionable target for change.

This study also has limitations. We sampled only 1 conference per region, per specialty, and for a single year. However, we selected the most popular or largest conference for each specialty. Data extraction excluded research abstract presentations, which accounted for a large component of some of the conferences, resulting in a small number of included sessions for these conferences. The data for the proportion of women in each specialty for the European region was estimated based on available data, which may not be an accurate representation. However, overall, 49% of physicians in Europe are women,^35^ which contrasts with the 26% of female speakers at conferences in the sample from this region in the present study, suggesting that women at European conferences are largely underrepresented. In addition, our study did not explore other factors that might be associated with these gender disparities. For example, women may be more likely to decline speaking invitations^36^ or may be less likely to put themselves forward for speaking opportunities.^37^ eAppendix 2 in the Supplement gives some factors associated with speaking invitations.

## Conclusions

In this cross-sectional study, the proportion of female speakers ranged from 5.8% to 74.5%; however, overall, women only represented 31.1% of all speakers. A total of 96% of conferences had at least 1 all-male panel, and one-third of all panel sessions were composed of only men. The study found that a higher proportion of women on planning committees was associated with a higher proportion of female speakers at conferences, suggesting that including women on planning committees can be used as a strategy for gender balance at future conferences.

## Notes

Supplement. **eAppendix 1.** Conference Name by Specialty and Region **eAppendix 2.** Factors Impacting Speaking Invitations at Conferences
