# Supplementary material for: Proportion of Female Speakers at Academic Medical Conferences Across Multiple Specialties and Regions
Source: JAMA Netw Open. 2020 Sep 28;3(9):e2018127. doi: 10.1001/jamanetworkopen.2020.18127 (PMC7522699; doi:10.1001/jamanetworkopen.2020.18127)
Supplement: Supplement. — eAppendix 1. Conference Name by Specialty and Region eAppendix 2. Factors Impacting Speaking Invitations at Conferences [file jamanetwopen-e2018127-s001.pdf]

Supplementary Online Content

Arora A, Kaur Y, Dossa F, Nisenbaum R, Little D, Baxter NN. Proportion of female speakers at academic medical conferences across multiple specialties and regions. *JAMA Netw Open*. 2020;3(9):e2018127. doi:10.1001/jamanetworkopen.2020.18127

eAppendix 1. Conference Name by Specialty and Region
eAppendix 2. Factors Impacting Speaking Invitations at Conferences

This supplementary material has been provided by the authors to give readers additional information about their work.

© 2020 Arora A et al. *JAMA Network Open.*

Appendix 1- Conference name by specialty and region

	**Conferences by Region** (Conference length in days)				
**Speciality**	**Australasia**	**Canada**	**Europe**	**United Kingdom**	**United States of America**
Anesthesiology	ANZ College of Anesthetists 2018 Annual Conference (5)	Canadian Anaesthesiologists' Society 2017 meeting (4)	Euroanaesthesia 2018 (3)	The Association of Anaesthetists of GBI Annual Congress 2018 (3)	Anesthesiology 2018 (5)
Cardiology	66th Annual Scientific meeting of the Cardiac Society of ANZ (3)	2017 Canadian Cardiovascular Congress (4)	European Society of Cardiology Congress 2018 (5)	British Cardiovascular Society Annual Conference 2018 (2)	American College of Cardiology 2018 (4)
Cardiothoracic surgery	27th Annual Congress of the Association of Thoracic and Cardiovascular Surgeons of Asia (4)	Canadian Surgery Forum 2017 (3)	25th European Society of Thoracic Surgeons Meeting (4)	2017 Society for Cardiothoracic Surgery in GBI Annual Meeting and Cardiothoracic forum (3)	American Association for Thoracic Surgery 98th Annual Meeting (4)
Colorectal surgery	6th Combined ANZ Colorectal Surgical Meeting 2017 (2)	Canadian Surgery Forum 2017 (3)	European Society of Coloproctology Thirteen Scientific and Annual Meeting 2018 (3)	Association of Coloproctology of Great Britain and Ireland Annual Meeting 2018 (3)	European Society of Coloproctology 13th Scientific & Annual Meeting (3)
Dermatology	51st Annual Scientific Meeting of the Australasian College of Dermatologists (4)	94th Canadian Dermatology Association Annual Conference (4)	European Academy of Dermatology and Venerology Congress 2018 (5)	97th Annual Meeting of British Association of Dermatologists (3)	2017 American Academy of Dermatology Annual Meeting (5)
Emergency medicine	34th Annual Scientific Meeting of the Australasian College for Emergency Medicine (5)	Canadian Association of Emergency Physicians Conference 2018 (4)	European Emergency Medicine Congress 2018 (5)	Royal College of Emergency Medicine Annual Scientific Conference 2017 (3)	American College of Emergency Physicians 2018 (4)
Endocrinology	Annual Endocrine Society of Australia Seminar 2018 (3)	20th Diabetes Canada/CSEM Professional Conference (4)	20th European Congress of Endocrinology (4)	Society for Endocrinology BES 2017 (3)	American Association of Clinical Endocrinologists 27th Annual Scientific & Clinical Congress (5)
Family medicine	RACGP Conference 2017 (3)	Family Medicine Forum 2017 (4)	23rd WONCA Europe Conference (4)	RCGP Annual Primary Care Conference and Exhibition 2018 (3)	AAFP Family Medicine Experience 2018 (5)
Gastroenterology	GESA's Australian Gastroenterology Week 2017 (3)	Canadian Digestive Diseases Week 2018 (4)	United European Gastroenterology Week 2018 (5)	British Society of Gastroenterology Annual Meeting 2018 (4)	Digestive Disease Week 2018 (4)
Geriatrics	50th Australian Association of Gerontology Conference (3)	CGS 37th Annual Scientific Meeting (3)	13th International Congress of the European Union Geriatric Medicine Society (3)	British Geriatrics Society Spring Meeting 2017 (3)	American Geriatrics Society 2018 Annual Scientific Meeting (3)
Neurology	Australian and New Zealand Association of Neurologists 2018 Annual Scientific Meeting (4)	Canadian Neurological Sciences Federation 53rd Congress (4)	European Academy of Neurology Congress 2018 (4)	Association of British Neurologists 2018 Annual Meeting (3)	American Academy of Neurology 2018 Annual Meeting (7)
Neurosurgery	Neurosurgical Society of Australasia 74th Annual Scientific Meeting (3)	Canadian Neurological Sciences Federation 53rd Congress (4)	European Association of Neurosurgical Societies 2018 (5)	Society of British Neurological Surgeons Spring Meeting 2018 (3)	American Association of Neurological Surgeons 2018 Annual Scientific Meeting (5)
Obstetrics/Gynecology	Royal Australian and New Zealand College of Obstetricians and Gynaecologists Annual Scientific Meeting 2018 (4)	Annual Clinical and Scientific Conference 2017 (4)	Congress of the European Society of Gynecology 2017 (4)	2017 Royal College of Obstetricians and Gynaecologists World Congress (3)	American College of Obstetricians and Gynecologists 2017 Annual Clinical and Scientific Meeting (4)
Oncology	Medical Oncology Group of Australia 2018 Annual Scientific Meeting (3)	Canadian Association of Medical Oncologists 2018 Annual Scientific Meeting (1)	European Society for Medical Oncology Congress 2018 (5)	2018 National Cancer Research Institute Cancer Conference (3)	2018 American Society of Clinical Oncology Annual Meeting (5)
Orthopedic surgery	Australian Orthopedic Association 2017 Annual Scientific Meeting (5)	The 2017 COA/CORS/CORA Annual Meetings (4)	18th European Federation of National Associations of Orthopaedics and Traumatology Annual Congress (3)	British Orthopaedic Association Annual Congress 2017 (4)	American Academy of Orthopaedic Surgeons 2018 Scientific Meeting (5)
Pathology	Royal College of Pathologists of Australasia 2018 Annual Scientific Meeting (3)	CAP-ACP 2018 Annual Meeting (4)	29th European Congress of Pathology (5)	10th Joint Meeting of the British Division of the International Academy of Pathology and the Pathological Society of Great Britain & Ireland (4)	College of American Pathologists 2018 (5)
Pediatrics	Pediatric Society of New Zealand 69th Annual Scientific Meeting 2017 (4)	Canadian Pediatric Society 94th Annual Conference (4)	European Academy of Paediatrics 2017 (4)	Royal College of Paediatrics and Child Health Conference and Exhibition 2018 (3)	American Academy of Pediatrics National Conference and Exhibition 2017 (4)
Psychiatry	Royal Australian and New Zealand College of Psychiatrists 2018 Congress (5)	Canadian Psychiatric Association 67th Annual Conference (3)	25th European Congress of Psychiatry (3)	Royal College of Psychiatrist Conference 2018 (5)	American Psychiatric Association 2017 Annual Meeting (5)
Radiology	Royal Australian and New Zealand College of Radiologists Annual Scientific Meeting 2017 (5)	Canadian Association of Radiologists Annual Scientific Meeting 2018 (4)	European Congress of Radiology 2018 (5)	The British Institute of Radiology Annual Congress 2017 (2)	American College of Radiology Annual Meeting 2018 (5)
Urology	Urological Society of Australia and New Zealand 2017 Annual Scientific Meeting (5)	Canadian Urological Association 73rd Annual Meeting (4)	European Association of Urology 2018 (5)	The British Association of Urological Surgeons 2017 Annual Meeting (3)	American Urological Association 2018 (4)

© 2020 Arora A et al. *JAMA Network Open.*

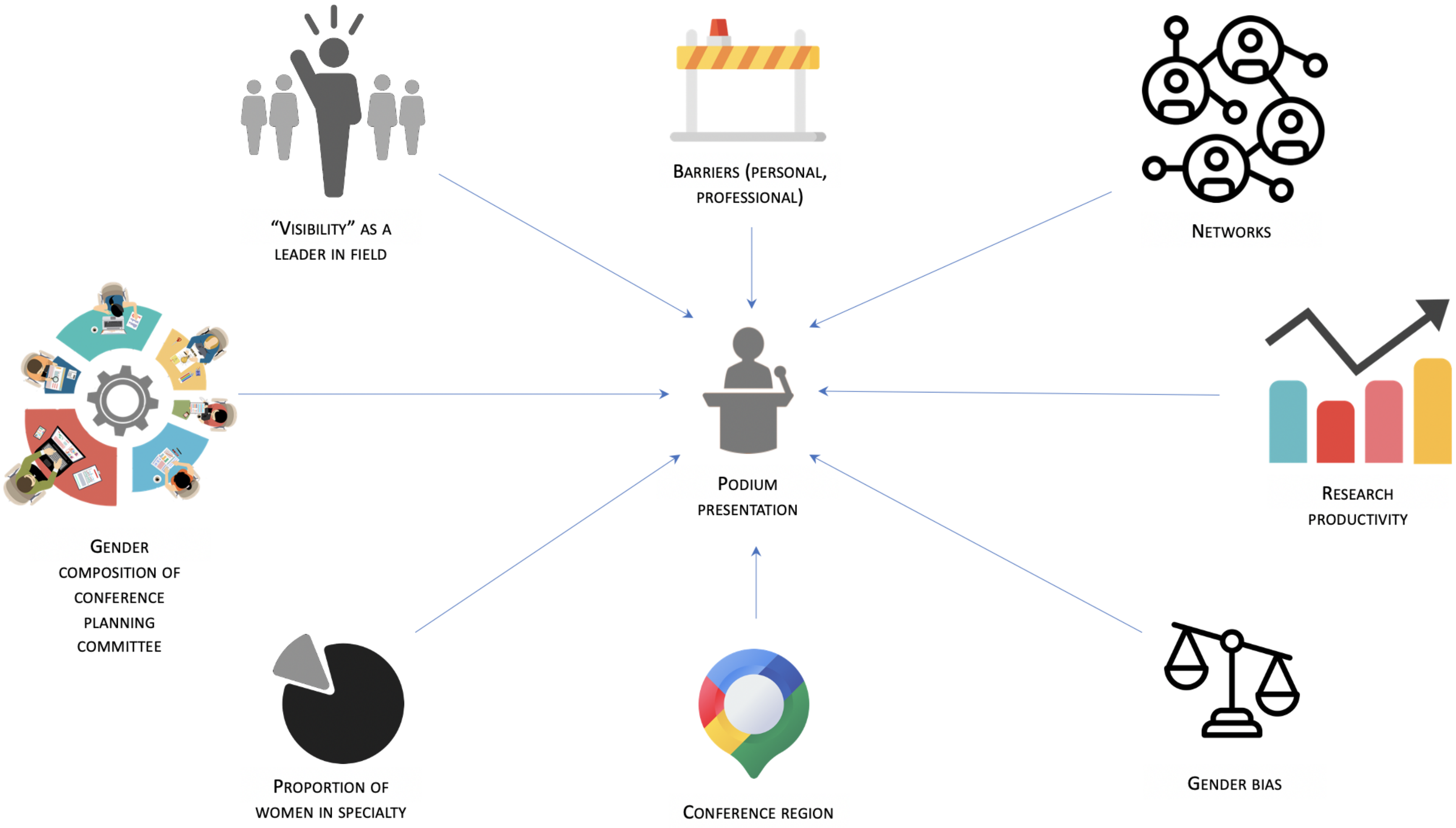

Appendix 2: Factors impacting speaker invitations at conferences

© 2020 Arora A et al. *JAMA Network Open.*
